# Accelerated hepatocellular carcinoma development in *CUL4B* transgenic mice

**DOI:** 10.18632/oncotarget.3829

**Published:** 2015-04-14

**Authors:** Jupeng Yuan, Baichun Jiang, Aizhen Zhang, Yanyan Qian, Haining Tan, Jiangang Gao, Changshun Shao, Yaoqin Gong

**Affiliations:** ^1^ Key Laboratory of Experimental Teratology, Ministry of Education, Institute of Molecular Medicine and Genetics, Shandong University School of Medicine, Jinan, China; ^2^ Key Laboratory of Experimental Teratology, Ministry of Education, Shandong University School of Life Science, Jinan, China

**Keywords:** CUL4B transgenic mice, DEN, HCC, ROS, proliferation

## Abstract

Cullin 4B (CUL4B) is a component of the Cullin 4B-Ring E3 ligase (CRL4B) complex that functions in proteolysis and in epigenetic regulation. CUL4B possesses tumor-promoting properties and is markedly upregulated in many types of human cancers. To determine the role of CUL4B in liver tumorigenesis, we generated transgenic mice that expressed human *CUL4B* in livers and other tissues and evaluated the development of spontaneous and chemically-induced hepatocellular carcinomas. We observed that *CUL4B* transgenic mice spontaneously developed liver tumors at a high incidence at old ages and exhibited enhanced DEN-induced hepatocarcinogenesis. There was a high proliferation rate in the livers of *CUL4B* transgenic mice that was accompanied by increased levels of Cdk1, Cdk4 and cyclin D1 and decreased level of p16. The transgenic mice also exhibited increased compensatory proliferation after DEN-induced liver injury, which was accompanied by activation of Akt, Erk, p38 and NF-κB. We also found that Prdx3 was downregulated and that DEN induced a higher level of reactive oxygen species in the livers of transgenic mice. Together, our results demonstrate a critical role of CUL4B in hepatocarcinogenesis in mice.

## INTRODUCTION

Liver cancer is among the most lethal and prevalent cancers worldwide [1, 2]. Liver cancer can be classified into hepatocellular carcinoma (HCC), intrahepatic bile duct carcinoma (cholangiocarcinoma), hepatoblastoma, bile duct cystadenocarcinoma, haemangiosarcoma and epitheliod haemangioendothelioma, with HCC accounting for 83% of all cases [3]. Chronic hepatitis B or C viral infection and exposure to genotoxic and cytotoxic chemicals, which usually cause chronic liver injury and inflammation, have been identified as the major risk factors of liver cancer [4].

*CUL4B* gene belongs to cullin family, which consists of eight members, *CUL1*, *CUL2*, *CUL3*, *CUL4A*, *CUL4B*, *CUL5*, *CUL7* and *PARC* [5]. The protein products of this family are important components of Cullin-RING E3 ubiquitin ligase complexes (CRLs) [6]. CRLs are the largest known class of E3 ubiquitin ligase family in eukaryote [6], and can ubiquitinate a wide array of substrates involved in diverse cellular processes, including cell cycle, gene expression, signal transduction, DNA damage response, chromatin remodeling, and embryonic development [7].

CUL4B expression is markedly upregulated in various human cancers [8-10]. Recently, we demonstrated that CRL4B can catalyze H2AK119 monoubiquitination and, in cooperation with PRC2, promote epigenetic silencing of tumor suppressors, leading to increased degree of malignancy *in vitro* and *in vivo* [8]. We found that CRL4B can also coordinate with SUV39H1/HP1/DNMT3A in promoting DNA methylation-based epigenetic silencing of tumor suppressors [9]. All these findings suggested that CUL4B played important roles in tumorigenesis. However, the role of CUL4B in hepatocarcinogenesis remains to be determined.

To clarify the roles of CUL4B in liver tumorigenesis, we generated transgenic mice expressing human *CUL4B* under the control of the *CMV* promoter and examined the development of spontaneous and chemically-induced hepatocellular carcinomas. Our results show that *CUL4B* transgenic mice spontaneously developed liver tumors and greatly accelerated DEN-induced hepatocellular carcinoma development.

## RESULTS

### Generation of transgenic mice overexpressing *CUL4B* under the control of the CMV promoter

**Figure 1:**
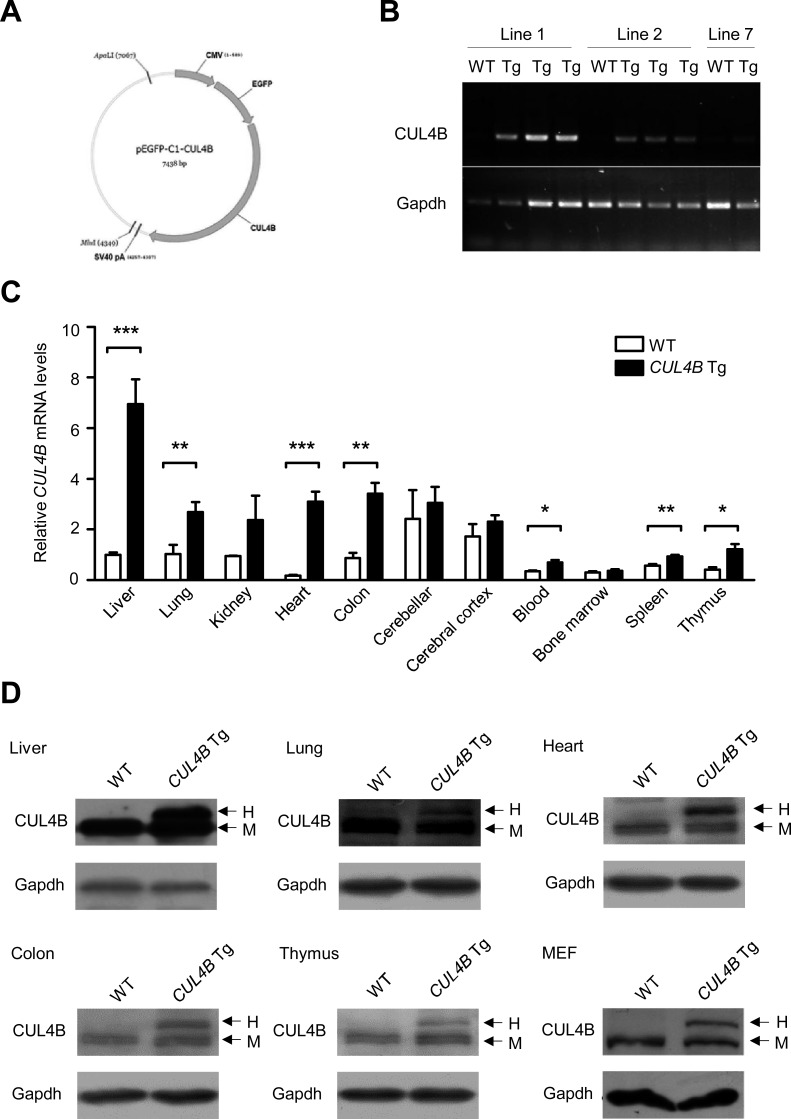
Expression of CUL4B in *CUL4B* transgenic mice **A.** The schematic of the construct used for generation of *CUL4B* transgenic mice. **B.** Expression of human *CUL4B* mRNA (transgenic) extracted from the tails of *CUL4B* transgenic mice assessed by RT-PCR using genotyping primers. **C.** Expression of both human (transgenic) and mouse (endogenous) *CUL4B* mRNA in various tissues of *CUL4B* transgenic and littermate control mice assessed by real-time RT-PCR (*n* = 5). **D.** Expression of human (transgenic) and mouse (endogenous) CUL4B protein in various tissues and mouse embryonic fibroblasts (MEFs) of *CUL4B* transgenic and littermate control mice was assessed by Western blotting. Representative results from one pair of animals are shown. Gapdh was used as a loading control. The upper bands represent the human (transgenic) CUL4B, whereas the lower bands represent the mouse (endogenous) Cul4b. H: human EGFP-CUL4B fusion protein; M: endogenous mouse Cul4b protein. Data were evaluated statistically by a two-tailed unpaired t test. Values are given as the mean ± SE. *: *p* < 0.05, **: *p* < 0.01, ***: *p* < 0.001.

To investigate the role of *CUL4B* in hepatocarcinogenesis, we first generated three lines of transgenic mice harboring a human *CUL4B* cDNA as well as *EGFP* cDNA under the control of the *CMV* promoter (Figure 1A, 1B and Figure S1A). Expression pattern of *CUL4B* (both human and mouse) in the tissues of transgenic mice line 1 was examined by real-time RT-PCR. The results showed that *CUL4B* was highly expressed in liver, lung, heart, colon, blood and thymus of this transgenic line (Figure 1C). We further examined the protein of human and mouse CUL4B in those tissues by Western blotting, which could distinguish between human and mouse CUL4B proteins because human CUL4B was fused with EGFP. We were able to detect the EGFP-CUL4B fusion protein in liver, lung, heart, colon and thymus, as well as embryonic fibroblasts (MEFs) of *CUL4B* transgenic mice but not in those of WT mice (Figure 1D). In addition, the green fluorescence was also observed in the toes of *CUL4B* transgenic mice (Figure S1B). These data indicated that human CUL4B was overexpressed in various tissues, including liver, of *CUL4B* transgenic mice.

### Aged *CUL4B* transgenic mice developed spontaneous liver tumors

**Figure 2:**
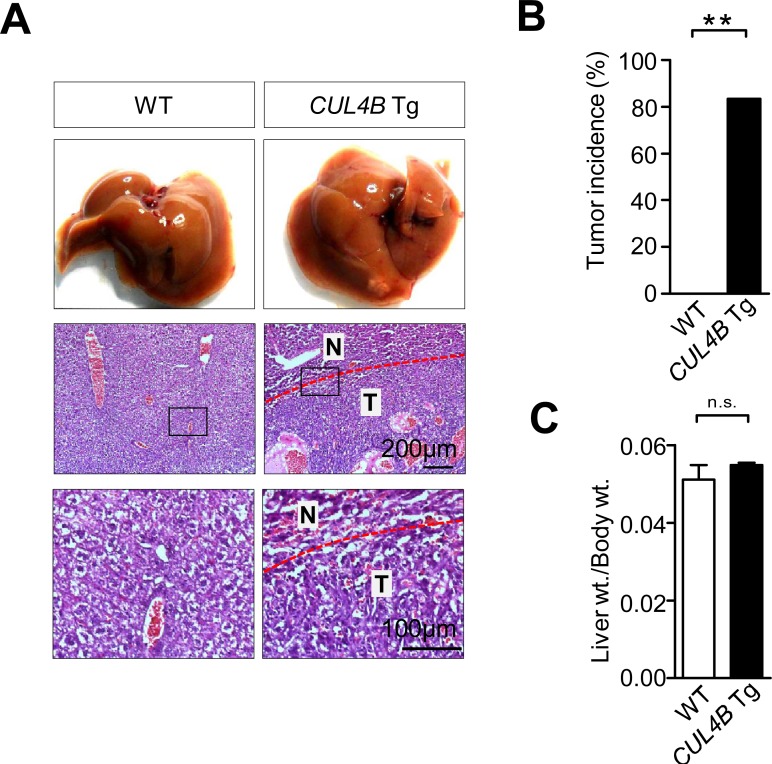
Aged *CUL4B* transgenic mice developed spontaneous liver tumors **A.** Representative photographs of gross morphology and histological analysis of livers from male *CUL4B* transgenic and littermate control mice at 2 years. N: non-tumor; T: tumor. **B.** The incidence of liver tumors in male *CUL4B* transgenic and littermate control mice at 2 years. **C.** The liver/body weight ratios in aged mice (*n* = 5). The incidence of liver tumors was analyzed by the Chi-square test, and the liver/body weight ratio was evaluated by a two-tailed unpaired t test. Values are given as the mean ± SE. *: *p* < 0.05, **: *p* < 0.01, ***: *p* < 0.001.

The body and liver of *CUL4B* transgenic mice weighed in normal range at 2-month-old (data not shown), and the liver histology was unremarkable (Figure S2). However, when the *CUL4B* transgenic mice were examined when they were 24-month-old, hepatocarcinomas were detected in 4 of 5 mice (80%), while it was not detected in littermate control mice (0/5) (Figure 2A, 2B). The liver/body weight ratio was not changed because of the small sizes of the tumors (Figure 2C). These data suggested that *CUL4B* transgenic mice were predisposed to the development of hepatocarcinogenesis.

### Enhancement of DEN-induced hepatocarcinogenesis in *CUL4B* transgenic mice

**Figure 3:**
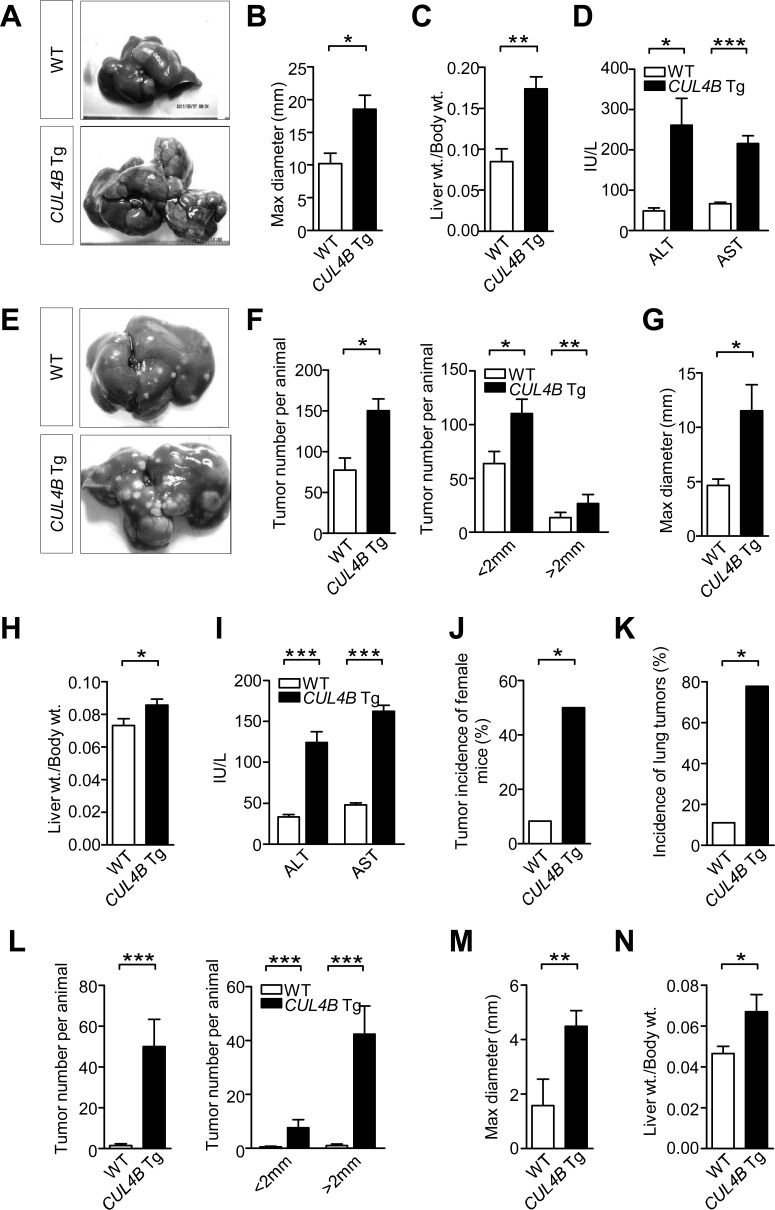
Enhancement of DEN-induced hepatocarcinogenesis in *CUL4B* transgenic mice **A.** Representative photographs of gross morphology of livers from male *CUL4B* transgenic and littermate control mice at 50 weeks after DEN administration and PB promotion. **B**-**D.** Determination of maximum tumor diameter **B**, liver/body weight ratios **C,** and levels of serum ALT and AST **D,** for *CUL4B* transgenic and littermate control mice at 50 weeks after DEN administration and PB promotion (*n* = 9). **E.** Representative photographs of gross morphology of livers from male *CUL4B* transgenic and littermate control mice at 24 weeks after DEN administration and PB promotion. **F**-**I.** Determination of the liver tumor numbers **F**, maximum (Max) tumor diameter **G**, liver/body weight ratios **H,** and levels of serum ALT and AST (I) for *CUL4B* transgenic and littermate control mice at 24 weeks after DEN administration and PB promotion (*n* = 7). **J.** The incidence of liver tumors in female *CUL4B* transgenic and littermate control mice at 50 weeks after DEN administration and PB promotion (*n* = 12). **K.** The incidence of lung metastasis in male *CUL4B* transgenic and littermate control mice at 50 weeks after DEN administration and PB promotion (*n* = 9). **L**-**N.** Determination of the liver tumor numbers **L**, maximum (Max) tumor diameter **M** and liver/body weight ratios **N.** in *CUL4B* transgenic and littermate control mice at 24 weeks after DEN treatment alone (*n* = 8). The incidence of liver tumors of female mice and the incidence of lung metastasis were analyzed by the Chi-square test, while the others were evaluated statistically by a two-tailed unpaired t test. Values are given as the mean ± SE. *: *p* < 0.05, **: *p* < 0.01, ***: *p* < 0.001.

DEN, a chemical carcinogen, was often used in combination with phenobarbital (PB), a tumor promoter, to induce liver cancer [11]. *CUL4B* transgenic and littermate control male mice were injected with DEN (10 mg/Kg) at postnatal day 14, and were administered with PB in the drinking water (0.025%) starting at 3-week-old. At 50 weeks after DEN treatment, all *CUL4B* transgenic mice and wild-type mice developed tumors, but the tumor masses in transgenic mice were significantly larger (Figure 3A, 3B). The liver/body weight ratio was higher in *CUL4B* transgenic mice because of the increased tumor masses (Figure 3C). Levels of serum ALT and AST were also increased in *CUL4B* transgenic mice when compared to wild-type mice (Figure 3D). In order to determine whether *CUL4B* transgenic mice developed more tumors at an early stage, we also sacrificed mice at 24 weeks after DEN injection. At 24 weeks after DEN treatment, almost all the *CUL4B* transgenic and littermate control mice developed liver tumors (Figure 3E). However, the number of detectable liver tumors in *CUL4B* transgenic mice was significantly increased compared to that of littermate control mice (Figure 3F), and the tumors were larger in *CUL4B* transgenic mice than in littermate control mice (Figure 3G). The liver/body weight ratio was also increased in *CUL4B* transgenic mice (Figure 3H). Levels of serum ALT and AST were dramatically increased in *CUL4B* transgenic mice (Figure 3I). Histological analysis of tumor regions showed that proliferating (PCNA-positive) cells were more abundant in *CUL4B* transgenic mice than in littermate control mice (Figure S3A). The expression of β-catenin was also increased in *CUL4B* transgenic mice than in littermate control mice (Figure S3B). As lipid droplet accumulation precedes the onset of DEN-induced HCC development, we examined the lipid droplet accumulation in liver. Indeed, lipid droplet accumulation was more evident in *CUL4B* transgenic mice than in littermate control mice (Figure S3C).

DEN treatment led to a lower incidence of liver tumors in female mice than in male mice [12]. Female mice were also injected with DEN and were administered with PB. At 50 weeks after DEN treatment, the incidence of liver tumors of female littermate control mice is only 8.3% (1/12), while the incidence of female *CUL4B* transgenic mice increased to 50% (6/12) (Figure 3J), suggesting that overexpression of CUL4B strongly promoted DEN-induced hepatocarcinogenesis in both male and female mice.

Lung metastasis has been reported in DEN-induced rat or mouse models [13, 14]. Therefore we also examined the incidence of lung metastasis in *CUL4B* transgenic mice 50 weeks after DEN treatment. While only 11% (1/9) of littermate control male mice showed lung metastases, 77.8% (7/9) of *CUL4B* transgenic male mice exhibited lung metastases (Figure 3K and S4). Immunohistochemistry using AFP (a marker of hepatocellular carcinoma) and Napsin A (a marker of lung tumors) antibodies confirmed that the tumors were metastatic from liver instead of being primary (Figure S4C).

To further confirm the promoting effect of CUL4B on DEN-induced hepatocarcinogenesis, we next tested the tumorigenic effect of a single DEN injection without the use of PB as a promoter. At 24 weeks after DEN injection, while the littermate control mice developed only a few liver tumors, the number of detectable liver tumors of *CUL4B* transgenic mice was significantly increased (Figure 3L and S5A). Moreover, most of the liver tumors of *CUL4B* transgenic mice were larger than 2 mm, with the maximal tumor diameter of 4.5 mm, compared to 1.6 mm in littermate control mice (Figure 3M). The liver/body weight ratio of *CUL4B* transgenic mice was approximately 45% higher than that in littermate control mice (Figure 3N). Serum ALT and AST levels were also significantly increased in *CUL4B* transgenic mice (Figure S5B).

### Accelerated spontaneous cellular proliferation in *CUL4B* transgenic livers

**Figure 4:**
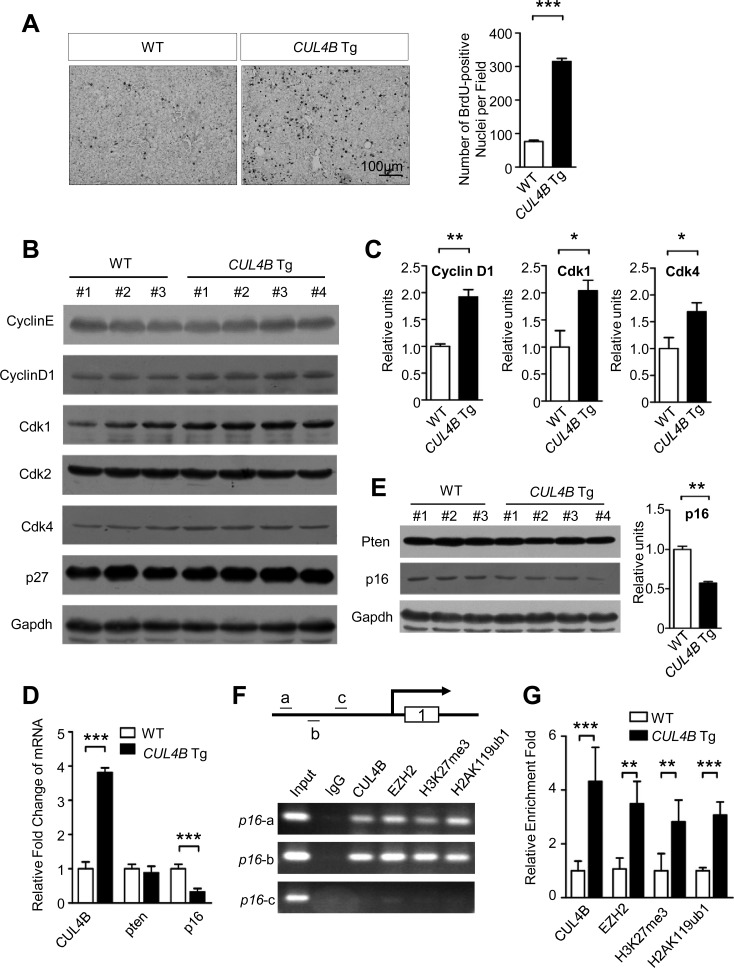
The effects of CUL4B overexpression on hepatocyte proliferation **A.** Proliferation of hepatocytes measured by BrdU incorporation assay in *CUL4B* transgenic mice compared to that in littermate control mice at the age of 2 weeks. **B.** Analysis of proteins involved in proliferation in livers of 2-week-old *CUL4B* transgenic and littermate control mice by Western blotting. Gapdh was used as a loading control. **C.** The intensity of each band was determined and its ratio to Gapdh was calculated. **D.** The RNA levels of epigenetically targeted genes of CUL4B, *p16* and *pten*, in the livers of *CUL4B* transgenic mice were measured by real-time RT PCR. **E.** The protein levels of p16 and pten in the livers of *CUL4B* transgenic mice were measured by Western blotting. The intensity of each band was determined and its ratio to Gapdh was calculated. **F.** ChIP experiments of the *p16* promoter (a, b and c) in mouse liver using the indicated antibodies. **G.** qChIP analysis of the *p16* promoter (fragment a) in liver of both WT and *CUL4B* transgenic mouse using the indicated antibodies. Results are presented as the fold change over control. Error bars represent the SD of three independent experiments. Values are given as the mean ± SD. *: *p* < 0.05, **: *p* < 0.01, ***: *p* < 0.001.

Previously we reported that CUL4B is critical for cell proliferation [8, 9, 15-17]. We next examined the spontaneous cell proliferation of hepatocytes in *CUL4B* transgenic mice by BrdU incorporation assay. The number of BrdU-positive cells in the livers of 2-week-old *CUL4B* transgenic mice was significantly increased compared with that in littermate control mice (Figure 4A), indicating that overexpression of CUL4B could greatly accelerate the cell proliferation of hepatocytes. To investigate the molecular mechanism underlying the increased hepatocyte proliferation in *CUL4B* transgenic mice, the proteins involved in cell cycle progression were examined in livers of 2-week-old *CUL4B* transgenic mice and littermate control mice. Western blotting showed that expressions of cyclin D1, Cdk1 and Cdk4 were upregulated (Figure 4B, 4C). These changes may have accounted for the increased cellular proliferation in the livers of the transgenic mice.

Previously we demonstrated that CUL4B can catalyze H2AK119 monoubiquitination and, in cooperation with PRC2, promote epigenetic silencing of tumor suppressors, leading to increased degree of malignancy [8]. To confirm whether enhanced hepatocarcinogenesis in *CUL4B* transgenic mice was mediated by the same mechanism, we examined the expressions of *p16* and *pten*, genes that are known to be repressed by CUL4B in some cancer cell lines, in the livers of *CUL4B* transgenic mice. While the expression of *pten* was not changed in livers of *CUL4B* transgenic mice, real-time RT-PCR assay showed that RNA level of *p16* was downregulated in *CUL4B* transgenic mice compared to that of littermate control mice (Figure 4D), suggesting that *p16* was also transcriptionally repressed by CUL4B in liver. Western blotting confirmed the p16 downregulation at protein level (Figure 4E). To investigate the molecular mechanism responsible for repression of *p16* by CUL4B, we performed chromatin immunoprecipitation (ChIP) assay to examine whether the promoter of *p16* was bound by CUL4B complex using the primers specific for the promoter of *p16* (Figure 4F). ChIP assays showed that CUL4B and EZH2 directly bind to the promoter of *p16*. H3K27me3 and H2AK119ub1, two histone markers of transcriptional repression, were also enriched in the same region (Figure 4F). Quantitative ChIP assays further showed that recruitments of CUL4B and EZH2 to the promoter of *p16* were increased with the overexpression of CUL4B (Figure 4G). Consistently, the levels of H3K27me3 and H2AK119ub1 at *p16* promoter were also increased, suggesting that CUL4B can transcriptionally repress *p16* expression by promoting H2AK119 monoubiquitination and H3H27 trimethylation.

As an E3 ligase, CRL4B targets various substrates for degradation. To investigate the possible roles of CUL4B substrates in accelerating cell proliferation of hepatocytes in *CUL4B* transgenic mice, we examined the expressions of known CUL4B substrates in the livers of *CUL4B* transgenic mice. The expression of cyclin E, a target of CUL4B, was not decreased in the liver of *CUL4B* transgenic mice compared to that of littermate control mice (Figure 4B). The expressions of other CUL4B substrates, including ERα, Topo I, WDR5, TSC2, Jab1 and p53, were also not changed (Figure S6). These data suggested that these molecules are probably not involved in CUL4B-induced cell proliferation of hepatocytes.

### Exacerbation of DEN-induced liver injury and increased compensatory proliferation in *CUL4B* transgenic mice

**Figure 5:**
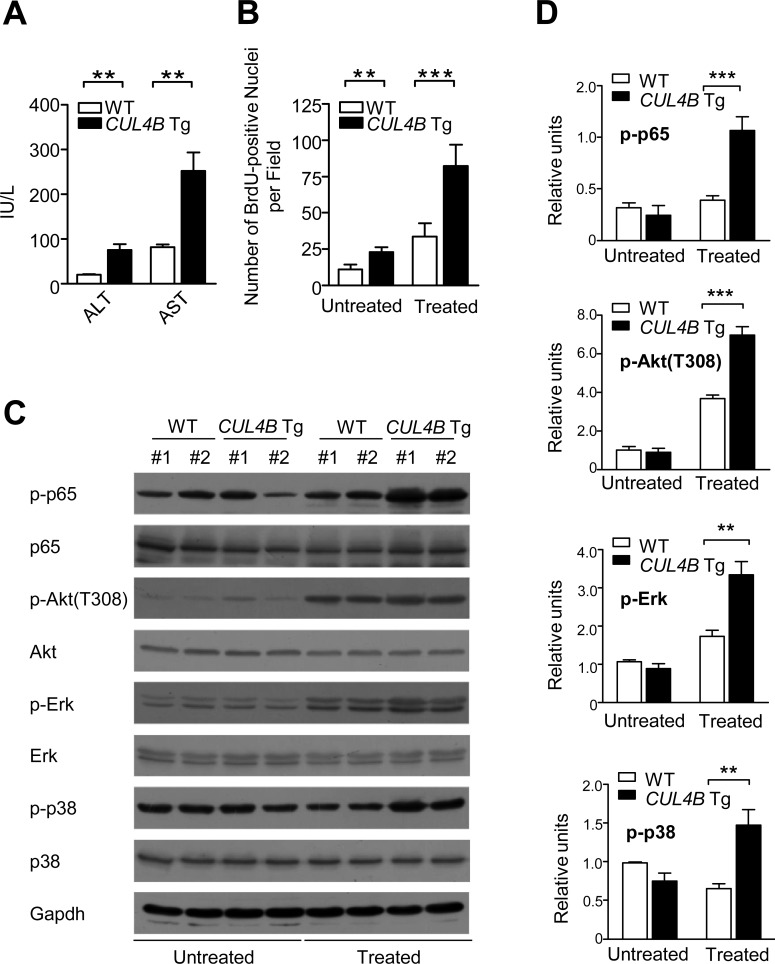
Overexpression of CUL4B exacerbates DEN-induced liver injury and causes increased compensatory proliferation **A.** Serum ALT and AST levels in *CUL4B* transgenic and littermate control mice with DEN injection (*n* = 6). **B.** Number of BrdU-positive cells in the livers of *CUL4B* transgenic and littermate control mice with or without DEN injection (*n* = 6). **C.** The levels of various phophorylated proteins in the livers of *CUL4B* transgenic and littermate control mice with or without DEN injection by Western blotting. Gapdh was used as a loading control. **D.** The intensity of each band was determined and its ratio to Gapdh was calculated. Values are given as the mean ± SD. *: *p* < 0.05, **: *p* < 0.01, ***: *p* < 0.001.

We next examined DEN-induced injury and the compensatory proliferation in order to gain insights into the enhancement in DEN-induced hepatocarcinogenesis in *CUL4B* transgenic mice. After DEN injection, serum ALT and AST levels increased significantly in *CUL4B* transgenic mice compared to those in littermate control mice (Figure 5A), indicating the presence of exacerbated liver damage.

To examine the compensatory growth after injury, we analyzed BrdU incorporation after DEN administration. As compared with littermate control mice, overexpression of CUL4B resulted in a substantial increase in the number of proliferating hepatocytes (Figure 5B). While p53 became activated and the level of γ-H2AX, which reflects DNA double-strand breaks, was increased after DEN injection, there was no significant difference between the transgenic mice and the controls (Figure S7). However, proliferation-driving pathways such as NF-κB, Akt, Erk and p38 signaling pathways were markedly upregulated in *CUL4B* transgenic mice after DEN administration (Figure 5C, 5D). Therefore, the increased proliferation may have accounted for the observed higher susceptibility of *CUL4B* transgenic mice to DEN induced hepatocarcinogenesis.

### Downregulation of Prdx3 and elevation of ROS in *CUL4B* transgenic livers

**Figure 6:**
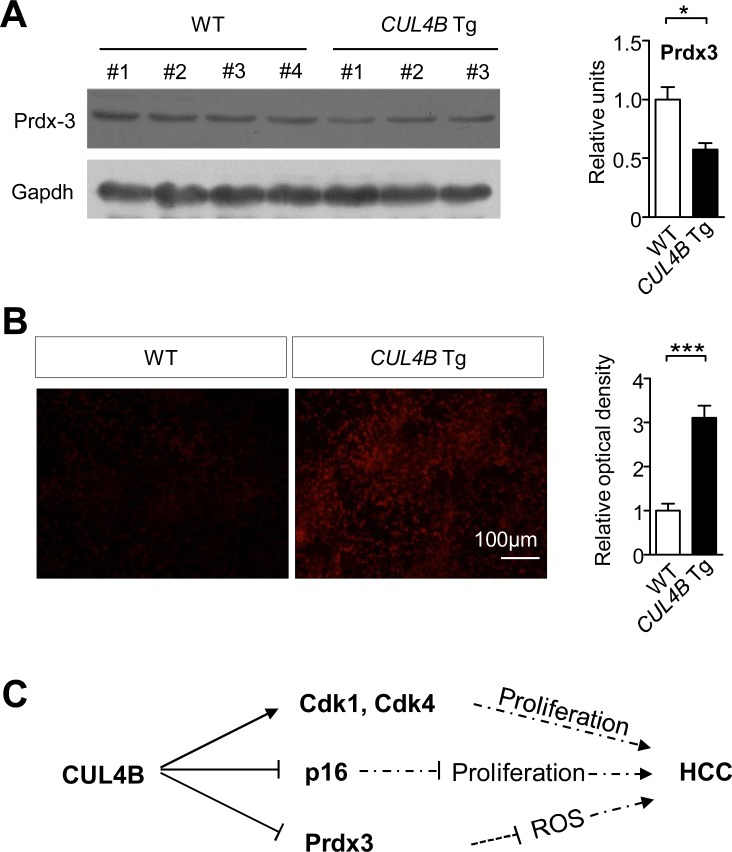
CUL4B overexpression decrease Prdx3 levels and increase ROS levels in livers **A.** Prdx3 level in the liver of *CUL4B* transgenic and littermate control mice was detected at 2 weeks by Western blotting. Gapdh was used as a loading control. The intensity of each band was determined and its ratio to Gapdh was calculated. **B.** Frozen liver sections prepared 24 hour after DEN injection were stained with 2 μM dihydroethidine hydrochloride for 30 min at 37°C. Cells stained positive for the oxidized dye were identified by fluorescence microscopy (*n* = 6). **C.** Graphic model as discussed in the text. Values are given as the mean ± SE. *: *p* < 0.05, **: *p* < 0.01, ***: *p* < 0.001.

Oxidative stress caused by DEN plays an important role in DEN-induced hepatocarcinogenesis [18-20]. We previously found that peroxiredoxin 3 (Prdx3), a scavenger of reactive oxygen species (ROS), was a substrate of CRL4B, and knockdown of CUL4B significantly decreased ROS levels [21]. To investigate whether the promoting effect of CUL4B on DEN-induced hepatocarcinogenesis was mechanistically mediated by increased ROS, we examined the Prdx3 level in the livers of *CUL4B* transgenic mice. Prdx3 was decreased in the livers of 2-week-old *CUL4B* transgenic mice compared to that in littermate control mice (Figure 6A). Accordingly, after DEN injection, the ROS levels in the liver of *CUL4B* transgenic mice were significantly increased compared to those in littermate control mice (Figure 6B). These findings suggest that decrease in the level of Prdx3 in *CUL4B* transgenic mice may have contributed to the increased level of oxidative liver damage upon DEN treatment.

## DISCUSSION

Although the main factors causing human HCC have been known for a long time, the precise knowledge about HCC pathogenesis and the mechanisms that regulate tumor development was limited. Animal models of human HCC can be used to identify specific genes and pathways involved in hepatocyte transformation, and to study the cellular and tissue context in which tumors develop [22, 23]. Chemically-induced HCC mouse models mimic the injury-fibrosis-malignancy cycle by administration of a genotoxic compound alone or followed by a promoting agent. A two-stage model is often used for inducing HCC, in which diethylnitrosamine (DEN) was used as an initiating agent and phenobarbital (PB) as a promoting agent [11]. To investigate the roles of CUL4B during hepatocarcinogenesis, we generated *CUL4B* transgenic mice and examined the development of spontaneous and DEN-induced hepatocarcinogenesis. We observed that overexpression of CUL4B could strongly promote spontaneous and DEN-induced hepatocarcinogenesis. We have also obtained data suggesting increased hepatocarcinogenesis in *CUL4B* transgenic mice could be mediated by increased proliferation and increased oxidative stress (Figure 6C).

Dysregulation of cullin activity has been shown to contribute to oncogenesis through the accumulation of oncoproteins or the excessive degradation of tumor suppressors [24]. CUL4A, which has the highest homolog with CUL4B, is overexpressed in primary breast cancers [25, 26] and hepatocellular carcinomas [27], and overexpression of CUL4A is associated with poor outcome in node-negative breast cancer [28]. Compared to CUL4A, CUL4B is less studied, and so far very few substrates of CUL4B CRL complexes have been identified, including estrogen receptor α (ERα) [29], Cyclin E [15], topoisomerase I (Topo I) [30], Peroxiredoxin 3 (Prdx3) [21], WDR5 [31], TSC2 [32], Jab1/CSN5 [33] and p53 [34]. However, the levels of most of these substrates in the livers of *CUL4B* transgenic mice were not found to differ from those in control mice, suggesting that cell type specific mechanism may operate in substrate degradation. Although several *Cul4b* knockout mouse models have been generated for the loss-of-function studies [17, 35, 36], gain-of-function studies of *CUL4B* using transgenic mice have not been reported. We therefore generated and analyzed a gain-of-function transgenic mouse model that overexpresses CUL4B. Overexpression of CUL4B in liver can greatly accelerate the cell proliferation of hepatocytes. We also showed that overexpression of CUL4B strongly promotes the development of DEN-induced liver tumors, as indicated by a two-stage model of DEN-PB induction or a single DEN injection without PB promotion. Furthermore, 2-year-old *CUL4B* transgenic mice developed liver tumor without any chemical induction, which further indicated that CUL4B overexpression could promote hepatocarcinogenesis.

To investigate the mechanism of DEN-induced hepatocarcinogenesis of *CUL4B* transgenic mice, we examined the early effects of DEN injection. Exacerbated hepatocyte damage was shown in *CUL4B* transgenic mice as indicated by serum ALT and AST levels. Hepatocyte proliferation was also increased in the livers of *CUL4B* transgenic mice, indicating increased compensatory growth after injury. Consistently, we observed that NF-κB, Akt, Erk and p38 pathways were significantly upregulated in the transgenic mice than in controls. On the other hand, Prdx3, a specific target of CUL4B E3 ligase, functions as a ROS scavenger. We previously showed that knockdown of CUL4B resulted in Prdx3 accumulation and significant decrease in cellular ROS production [21]. Consistent with these observations, CUL4B overexpression decreased Prdx3 level in livers, and increased ROS level after DEN induction.

Overall, overexpression of CUL4B strongly promotes spontaneous and DEN-induced hepatocarcinogenesis, which is probably due to increased hepatocyte proliferation and increased ROS level after liver damage. Thus, *CUL4B* transgenic mice may provide a useful animal model to study cancer development in the liver and broaden our knowledge of *CUL4B* as an oncogene. Blocking of CUL4B expression or activity would be a potent strategy to delay and/or prevent HCC development.

## MATERIALS AND METHODS

### Generation of *CUL4B* transgenic mice

For construction of a *CUL4B* transgene, an *EGFP* cDNA, the full-length human *CUL4B* cDNA and an SV40 polyadenylation signal were cloned into a mammalian expression vector containing the *CMV* promoter, giving rise to the *CMV-EGFP-CUL4B* transgene (Figure 1A). The transgene was released by *Apa*LI and *Mlu*I from the vector backbone and purified for pronuclear injection. Transgenic founders with CD-1(ICR) background were generated. PCR genotyping showed that three founders were obtained (Figure S1A). RT-PCR using the genotyping primers showed that the transgene were highly expressed in two lines, line 1 and line 2 (Figure 1B). Both lines were used in the following experiments and similar results were obtained, although only the results of line 1 were shown. All experiments involving animals were conducted in compliance with national regulations and by protocols approved by institutional animal care and use committee.

### PCR genotyping

Genomic DNA was extracted from mouse tails and used for genotyping by PCR analysis. For the genotyping of *CUL4B* transgenic mice, primers p01 (5′-TGGTCCTGCTGGAGTTCGTG-3′, binds to *EGFP* cDNA) and p02 (5′-GGCGGAGTGGTGCTGGTATT-3′, binds to *CUL4B* cDNA) were used to amplify the transgene (223 bp).

### Reverse transcription PCR and real-time RT-PCR

Total RNA was isolated using Trizol reagent (Invitrogen, Carlsbad, CA, USA), and treated with RQ1 RNase-Free DNase (Promega, Madison, WI, USA) to eliminate genomic DNA contamination. Freshly isolated RNA was reverse transcribed to generate cDNA using reverse transcriptase (Thermo Scientific, Rockford, IL, USA) following the manufacturer's recommendations. *CUL4B* gene was amplified by PCR using cDNA as template. Real-time RT-PCR was performed for quantitation of *CUL4B* mRNA using the Roche 480 instrument (Roche; Roche Diagnostics, Basel, Switzerland). The mRNA levels of *CUL4B* were measured by SYBR Green assay using LightCycler 480 SYBR Green I Master (Roche). Mice *Gapdh* was used as endogenous control. The sequences of the primers were for *Cul4b*, 5′-GCAACTGGAATAGAGGATGGA-3′ and 5′-TCTTTCTGTAGTGCTTGCTTGT-3′, for *Pten*, 5′-CTGCAGAGTTGCACAGTATCC-3′ and 5′-TAATATACATAGCGCCTCTGACTG-3′, for *p16*, 5′-GAACTCTTTCGGTCGTACCC-3′ and 5′-GCACGATGTCTTGATGTCCC-3′ and for *Gapdh*, 5′-AGGTCGGTGTGAACGGATTTG-3′ and 5′-TGTAGACCATGTAGTTGAGGTCA-3′. Four independent measurements per sample were performed. The quantified individual RNA expression levels were normalized to *Gapdh*.

### Western blotting

Total protein was extracted from liver tissues of *CUL4B* transgenic and littermate control mouse at different ages. Equal amount (50 μg) of total protein was immunoblotted by standard procedures. Primary antibodies used include: anti-CUL4B (Sigma, St Louis, MO, USA; 1:1,000), anti-CyclinD1 (Abcam, Hong Kong, China; 1:1,000), anti-CDK1 (Cell Signaling Technology, Beverly, MA, USA; 1:1,000), anti-CDK2 (CST; 1:1,000), anti-CDK4 (CST; 1:1,000), anti-p16 (Santa Cruz Biotechnology, Dallas, TX, USA; 1:500), anti-p27 (Santa Cruz; 1:1,000), anti-γ-H2AX (Millipore, Billerica, MD, USA; 1:1,000), anti-Rad51 (Santa Cruz; 1:1,000), anti-p-p53 (CST; 1:1,000), anti-p53 (Santa Cruz; 1:1,000), anti-p-p65 (CST; 1:1,000), anti-p65 (Santa Cruz; 1:1,000), anti-p-p38 (CST; 1:1,000), anti-p38 (CST; 1:1,000), anti-p-AKT(T308) (CST; 1:1,000), anti-AKT (CST; 1:1,000), anti-p-ERK (CST; 1:2,000), anti-ERK (CST; 1:1,000), anti-Cyclin E (Santa Cruz; 1:1,000), anti-Topo I (Santa Cruz; 1:1,000), anti-TSC2 (Santa Cruz, 1:1,000), anti-PTEN (Abcam, 1:1,000), anti-ERα (CST, 1:1,000), anti-JAB1 (Santa Cruz, 1:1,000), anti WDR5 (Abcam, 1:1,000) and anti-Prdx3 (Sigma; 1:1,000). Secondary antibodies include anti-mouse and anti-rabbit horseradish peroxidase (HRP) (Jackson ImmunoResearch, West Grove, PA, USA; 1:10,000). Chemiluminescence detection was performed by an ECL kit (Thermo). GAPDH was used as a loading control (Sigma; 1:5,000).

### Chromatin immunoprecipitation (ChIP)

ChIP was performed as described previously [37]. Briefly, fresh liver tissue was cut into small pieces, then cross-linked with 1% formaldehyde, sonicated, pre-cleaned, and incubated with 5-10 μg of antibody per reaction. After being mixed with magnetic beads for 2 hours, complexes were washed with low and high salt buffers, and the DNA was extracted and precipitated. The enrichment of the DNA template was analyzed by conventional PCR using primers specific for each target gene promoter. The sequences of the primers were for *p16*-a, 5′-GCAACAGGGAATGGAACT-3′ and 5′-AGGTATCTGGGCAGAAGG-3′ (−1800bp to −1400bp upstream of the TSS), for *p16*-b, 5′-CAAAGTCACATACTAGAGGGAA-3′ and 5′-GGGTCTTATAGAGCGGATT-3′ (−1400bp to −1000bp), and for *p16*-c, 5′-CTTCCCGCTTTCTCAATCTCC-3′ and 5′-CCCGGCTCTTCCTCTTTCC-3′ (−1000bp to −600bp). Antibodies used in ChIP assay were anti-CUL4B (Sigma), anti-EZH2 (BD, Franklin Lakes, NJ, USA), anti-H3K27me3 (Millipore) and anti-H2AK119ub1 (CST).

### Immunohistochemistry

Immunohistochemistry was performed as described previously [17]. Briefly, specimens were dissected and fixed in 4% paraformaldehyde at 4°C overnight, followed by cryo-section. Primary antibodies used include: anti-Cul4b (Sigma; 1:1,000), anti-PCNA (Abcam; 1:200), anti-AFP (Proteintech Group, Chicago, IL, USA; 1:100), anti-Napsin A (Proteintech; 1:100) and anti-β-Catenin (Santa Cruz; 1:200). Secondary antibodies include: anti-mouse and anti-rabbit horseradish peroxidase (HRP) (Jackson ImmunoResearch; 1:200). Negative controls were obtained by substituting the primary antibody with normal serum.

### BrdU incorporation

For labeling of cells in S phase, BrdU (Sigma) was injected intraperitoneally into mice with 100 mg per Kg body weight. Animals were sacrificed after 2 hours by cervical dislocation and the tissues were recovered in ice cold PBS and were fixed in 4% paraformaldehyde. Incorporation of modified nucleotide was detected by staining with an anti-BrdU primary antibody (Abcam; 1:100) and HRP-labeled secondary antibody (Jackson ImmunoResearch; 1:100).

### DEN administration

To induce HCC, DEN (10 mg/kg; Sigma) was injected intraperitoneally into 14-day-old mice. Parts of the mice were administered with phenobarbital (PB, Sigma) in the drinking water (0.025%) from the age of 3 weeks. Mice were sacrificed at 24 or 50 weeks after DEN treatment. Body and liver weights were recorded, and livers were removed and separated in individual lobes. Externally visible tumors (>0.5 mm) were counted and measured by stereomicroscopy.

### ROS measurement

Dihydroethidine hydrochloride (DHE, Sigma), was used to evaluate the levels of ROS. Mouse livers prepared 24 hour after DEN injection were frozen sectioned at 7 μm and stained with 2 μM DHE for 30 min at 37°C. Cells staining positively for the oxidized dye were identified by fluorescence microscopy.

### Statistical analysis

Data represented mean ± SE. Data from the two groups were evaluated statistically by a two-tailed unpaired t test using SPSS for Windows (version 13; SPSS Inc., Chicago, IL, USA) for any significant differences. A *p* value of less than 0.05 was considered statistically significant.

## SUPPLEMENTARY MATERIAL FIGURES


