# Supplementary material for: Accelerated hepatocellular carcinoma development in CUL4B transgenic mice
Source: Oncotarget. 2015 Apr 14;6(17):15209–21. doi: 10.18632/oncotarget.3829 (PMC4558146; doi:10.18632/oncotarget.3829)
Supplement: Supplementary file 1 [file oncotarget-06-15209-s001.pdf]

Accelerated hepatocellular carcinoma development in *CUL4B* transgenic mice

Supplementary Material

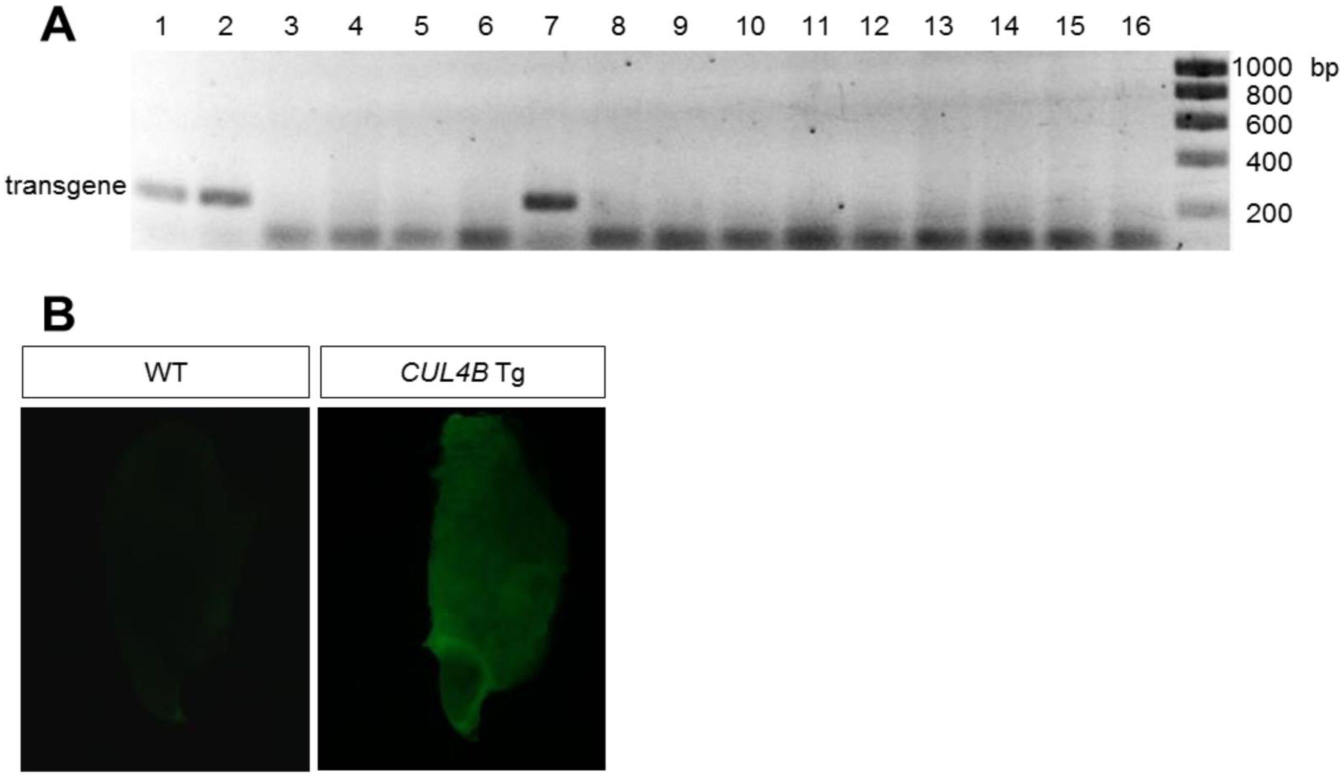

Figure S1. Characterization of *CUL4B* transgenic mice.

(A) PCR genotyping showed that three founders were obtained.

(B) The green fluorescence was observed in the toes of *CUL4B* transgenic mice.

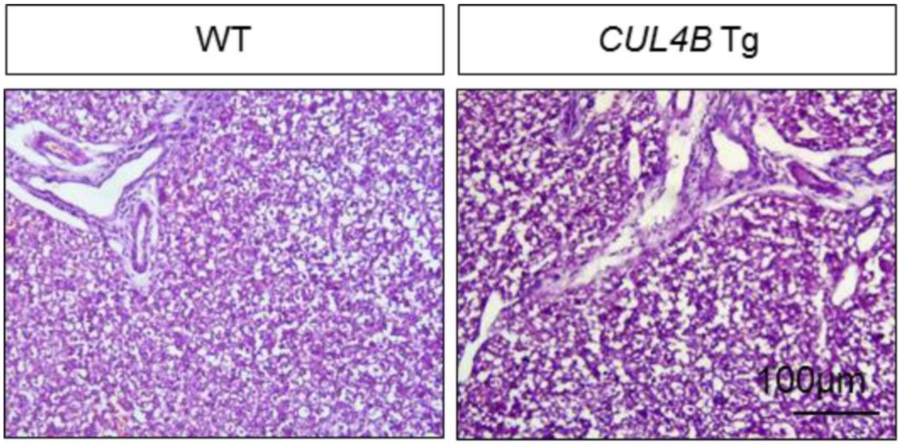

Figure S2. Histological analysis of the livers of *CUL4B* transgenic mice.

H&E-stained sections of the livers of *CUL4B* transgenic and littermate control mice at 2 months.

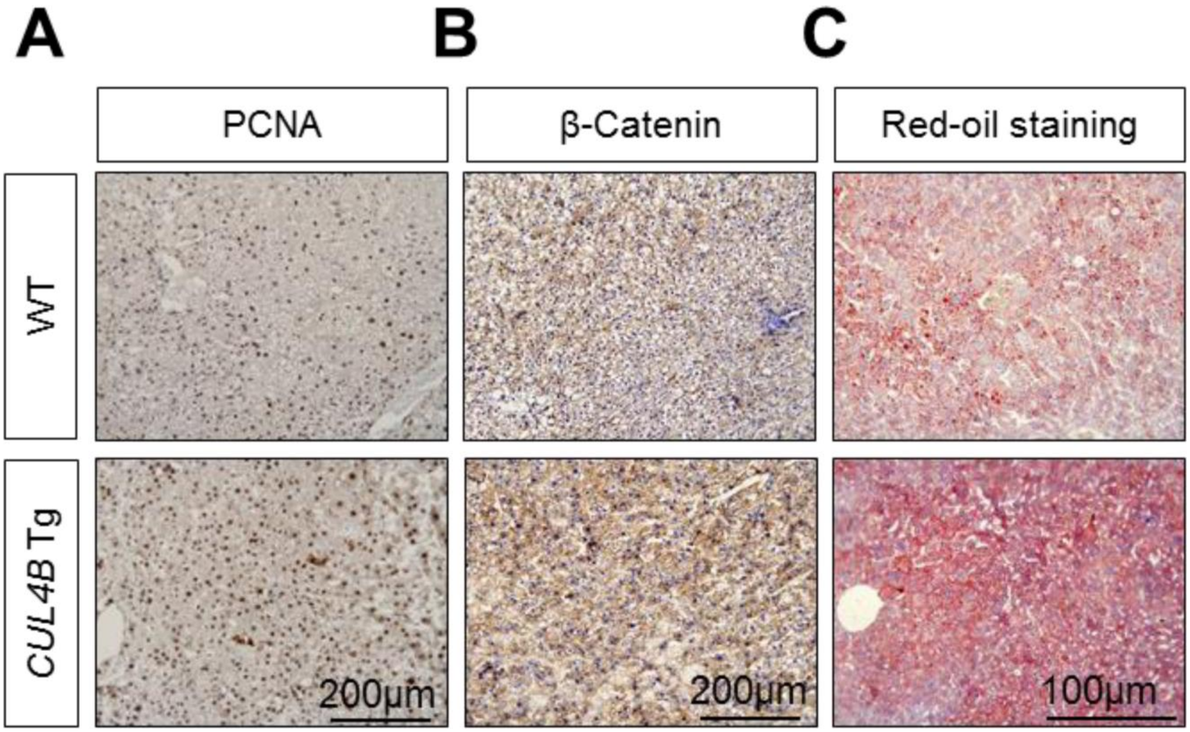

Figure S3. Histological analysis of the livers of DEN-treated *CUL4B* transgenic and littermate control mice at 24 weeks after DEN administration and PB promotion.

(A) PCNA immunostaining, (B) β-catenin immunostaining, (C) red-oil staining.

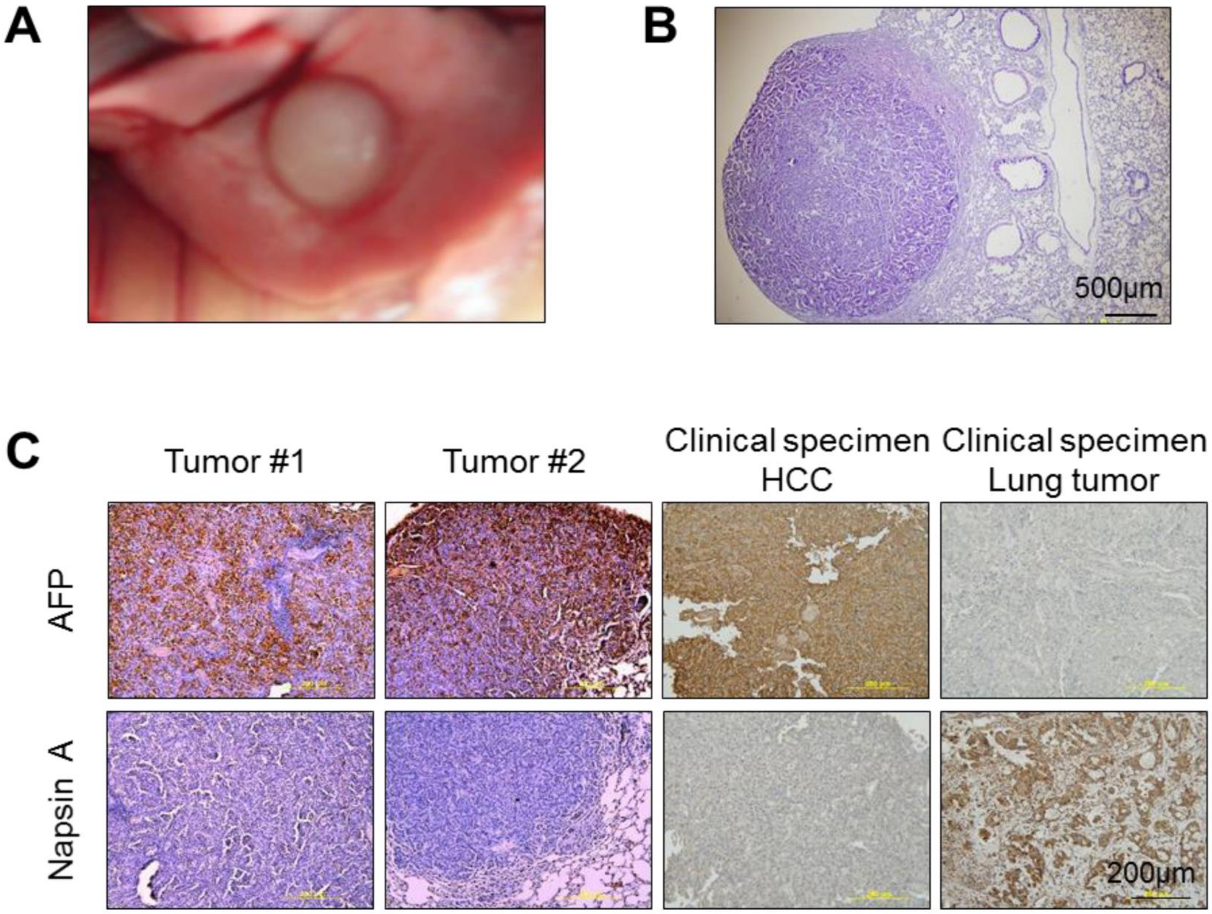

Figure S4. Lung metastasis in *CUL4B* transgenic mice treated with DEN induction and PB promotion.

(A) Representative gross photographs of lungs from male *CUL4B* transgenic mice at 50 weeks after DEN treatment.

(B) Histological analysis of the lungs of *CUL4B* transgenic mice were evaluated by H&E staining.

(C) Immunohistochemistry staining of metastasis tumors (tumor #1 and tumor #2) and clinical specimens with indicated antibodies. AFP served as a marker of hepatocellular carcinoma, while Napsin A was a marker of lung tumors. Clinical specimens including HCC and lung tumor served as the positive and negative control of AFP immunostaining and Napsin A immunostaining.

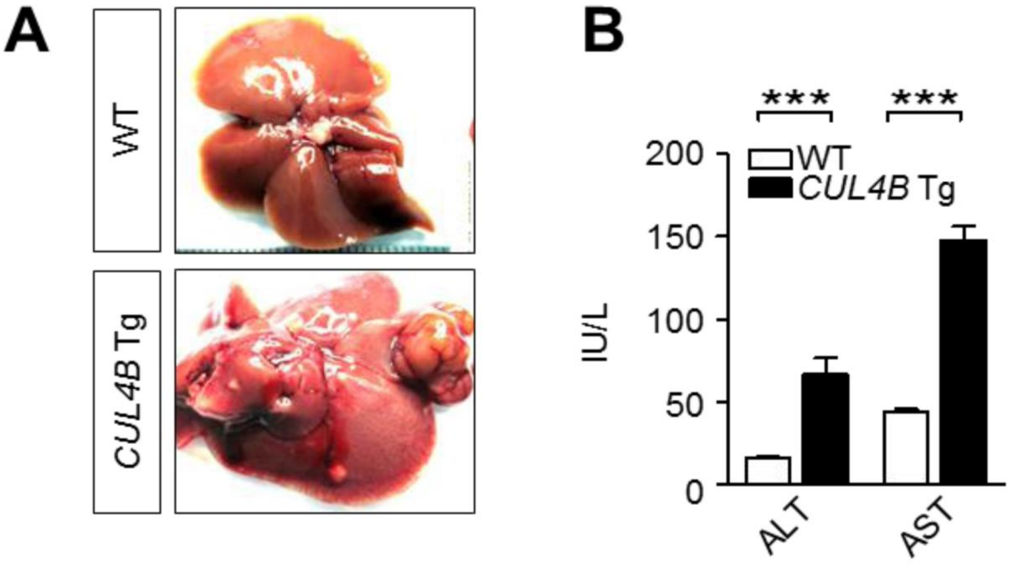

Figure S5. Tumor development in *CUL4B* transgenic mice treated with DEN induction without PB promotion.

(A) Representative gross photographs of livers from male *CUL4B* transgenic and littermate control mice at 24 weeks after DEN treatment alone.

(B) The levels of serum ALT and AST for *CUL4B* transgenic and littermate control mice (n=8). Values are given as the mean ± SE. *: $p<0.05$, **: $p<0.01$, ***: $p<0.001$.

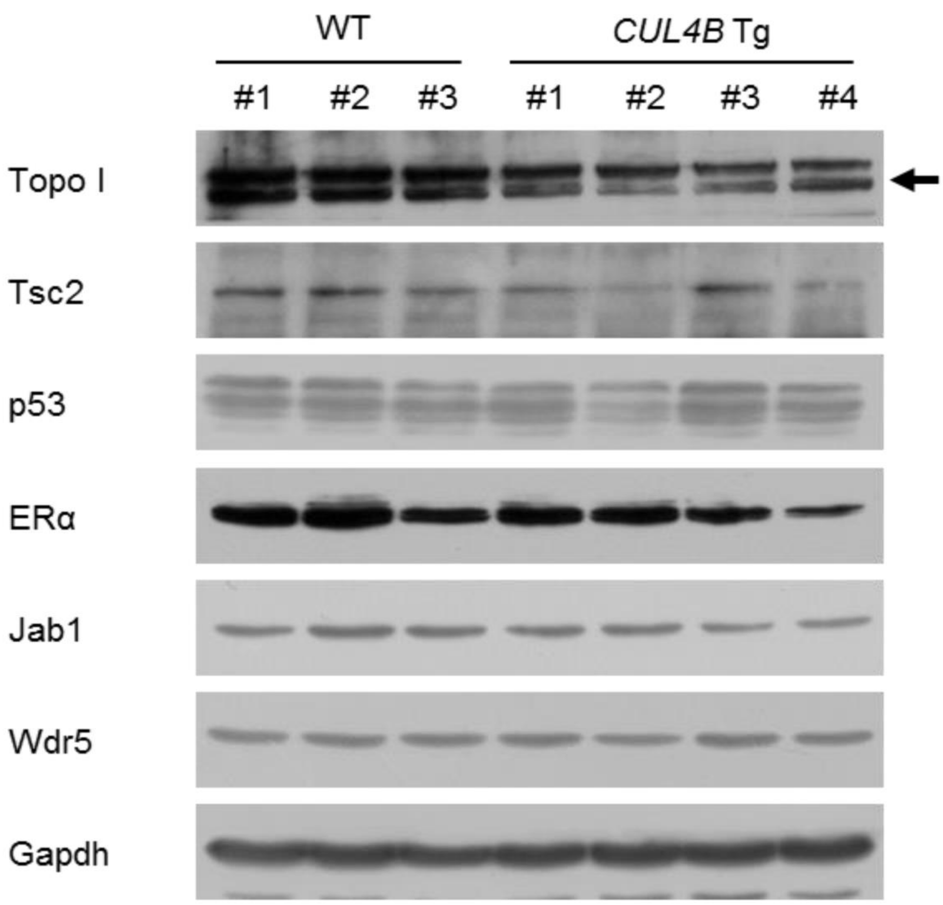

Figure S6. The status of substrates of CUL4B in *CUL4B* transgenic mice.

The substrates of CUL4B were measured by Western blotting using fresh liver tissues from *CUL4B* transgenic mice as well as their littermate control mice at the age of 2 weeks.

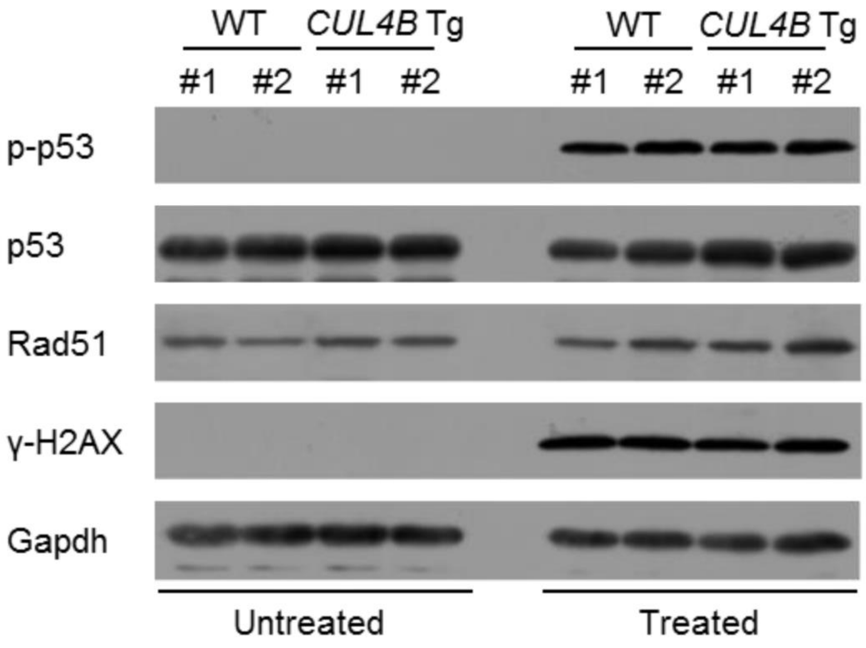

Figure S7. The expression of proteins involved in DNA damage-related pathways in *CUL4B* transgenic and littermate control mice by Western blotting.

The expression of protein was detected in the livers of *CUL4B* transgenic and littermate control mice with or without DEN injection by Western blotting. Gapdh was used as a loading control.
